# Preoperative Prognostic Nutritional Index Predict Survival in Patients With Resectable Esophageal Squamous Cell Carcinoma

**DOI:** 10.3389/fnut.2022.824839

**Published:** 2022-04-13

**Authors:** Zhiwei Zheng, Huide Zhu, Hongfu Cai

**Affiliations:** ^1^Department of Pharmacy, Cancer Hospital of Shantou University Medical College, Shantou, China; ^2^Department of Pharmacy, Fujian Medical University Union Hospital, Fuzhou, China

**Keywords:** prognostic nutritional index, esophageal squamous cell carcinoma, overall survival, prognosis, radical esophagectomy

## Abstract

**Background:**

Prognostic nutritional index (PNI) is one of the most important factors related to prognosis in many types of cancer. This study aimed to evaluate the PNI on predicting the overall survival (OS) in resectable esophageal squamous cell carcinoma (ESCC).

**Methods:**

A total of 165 patients with resectable ESCC were included in our retrospective study. PNI values before surgery were calculated for each patient [PNI = 10 × albumin (gr/dL) + 0.005 × total lymphocyte count (mm^3^)]. PNI cutoff value was selected by drawing receiver operating characteristics (ROC) curve, which used OS time as the endpoint. The Kaplan-Meier method and the Cox regression model of multivariate analysis were used to analyze the prognostic relationship between PNI and OS.

**Results:**

Among the 165 patients, 34 (20.6%) were women and 131 (79.4%) were men. The mean age was 62.67 ± 7.95 years, with the age range from 44 to 85 years. The average PNI was 46.68 ± 8.66. ROC curve showed that the best cutoff value was 43.85. All patients were divided into two groups: 72 patients (43.6%) were in the low PNI group (<43.85), while 93 patients (56.4%) were in the high PNI group (≥ 43.85). Univariate analysis demonstrated that PNI, tumor length, and T-stage and pathological stage were related to the prognosis of patients with ESCC (*P* <0.05). The Kaplan-Meier curve showed that the high PNI group has significantly increased OS compared to low PNI group (*p* = 0.01). Three-year OS rates were 57.5% in the low PNI group while 77.7% in the high PNI group. Univariate analysis showed that advanced pathological stage, large tumor length, and low PNI (separately, *p* < 0.05) were significant risk factors for shorter OS. Multivariate analysis showed that tumor length (*P* = 0.008) and PNI (*P* = 0.017) were independent prognostic factors in patients with resectable ESCC.

**Conclusion:**

PNI is a simple and useful predictive marker for the OS time in patients with radical esophagectomy.

## Introduction

Esophageal cancer (EC) is one of the most common malignant tumors in the world. This disease has a crude mortality rate of 7.8/100,000 in 2020, which represented 5.5% of all cancer deaths and ranked as the sixth most common cause of cancer death (1, 2). In China, EC is the fourth most common cause of mortality, with 30.1 deaths per 100,000 in 2020 (3). Despite advances in the treatment of esophageal squamous cell carcinoma (ESCC), radical surgical operation remains the first choice of treatment, but the overall survival (OS) remains poor. In Japan, the 5-year survival rate of esophageal cancer is 44.1% (4), while in China it is 30.3% in the same period (5). Hence, there is a continuing interest in looking for a simple and useful prognostic maker to identify patients with ESCC who are at greater risk.

Due to dysphagia, swallowing pain, eating obstruction, and tumor consumption, patients with esophageal cancer are prone to malnutrition (6). Recently, the prognostic nutritional index (PNI) has been reported to be a prognostic marker in various gastrointestinal cancer, such as gastric cancer and gastro-esophageal junction cancer (7–10). Until now, there are few studies focused on the relationship between PNI and OS in resectable ESCC. Besides, the best critical value of PNI for predicting cancer prognosis is different in many reports. Thus, the aim of this research was to evaluate the prognostic value of PNI in predicting OS with ESCC and validate the best critical cutoff value of PNI in ESCC.

## Materials and Methods

### Patients

From January 2017 to August 2020, a retrospective analysis was conducted in 165 patients with ESCC that underwent radical esophagectomy at the cancer hospital of Shantou University Medical College (Guangdong, China). All of the patients included in the analysis met the following criteria: (1) have ESCC confirmed by histopathology; (2) had surgery or preceded by adjuvant chemotherapy/radiotherapy before surgery; (3) have curative esophagectomy with R0 resection (*en bloc* resection with margins histologically free of disease); (4) have American Society of Anesthesiology (ASA) grade of 1-2. The ASA grade 1 was among 56 patients, which occupied 33.94% of our study group. The ASA grade 2 was among 109 patients, which occupied 66.06% of our study group.

Albumin and lymphocyte counts were collected using a routine blood test within one week before surgery. The patients' clinicopathological characteristics and pathological data were obtained from medical records.

### Follow-Up and Definitions

At our hospital, patients were followed up through telephone interviews and regularly received follow-up check-ups in the outpatient department. Recording of medical history, physical examination, blood routine, blood biochemistry, and CT scan of the chest were performed every 3 months for the first 2 years after surgery, then annually.

The last follow-up date was September 2021.

Overall survival was defined as the interval from the date of surgery to the date of cancer-related death or last contact.

The PNI was calculated using the following formula: PNI = 10 × albumin (gr/ dL) +0.005 x total lymphocyte count (mm^3^). The best cut-off value of PNI was selected by drawing the ROC curve used OS time as the endpoint.

### Statistical Analysis

The statistical was performed using IBM SPSS software version 21. The best cut-off value of PNI was selected by drawing the ROC curve, which used OS time as the endpoint. Then, the Youden index (sensitivity + specificity−1) was calculated. Independent sample *T*-test and one-way ANOVA were used for the comparison of measurement data.

Enumeration data were compared by the chi-square test. The Kaplan-Meier method and the Cox regression model of multivariate analysis were used to analyze the prognostic relationship between PNI and OS. *P* < 0.05 was considered statistically significant.

## Results

### The ROC Curve for an Optimal Cutoff Value

**Figure 1:**
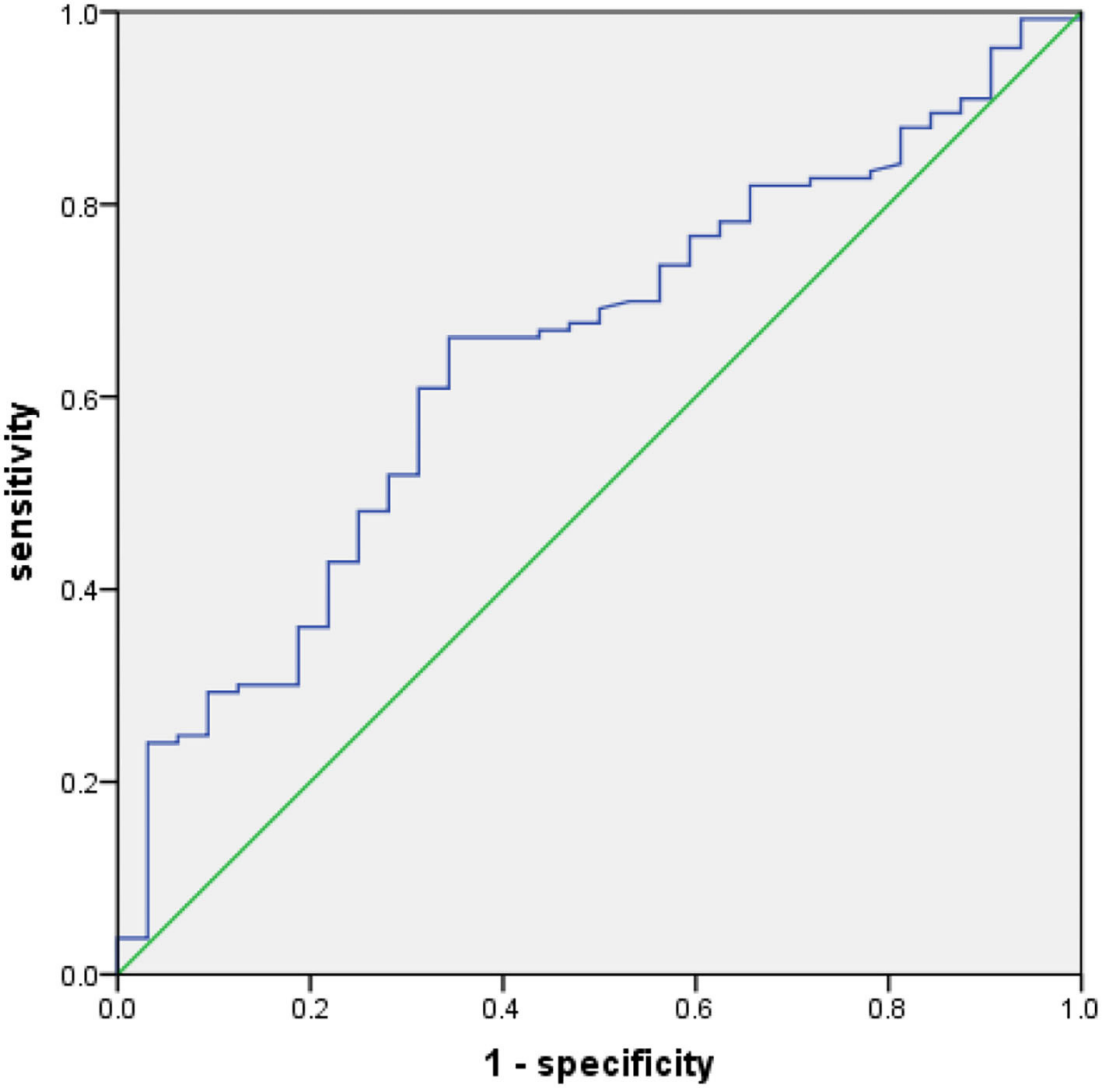
Receiver operating characteristics (ROC) for overall survival (OS) was plotted to calculate the best critical value for prognostic nutritional index (PNI).

The ROC curve was plotted as shown in Figure 1. When the PNI value was 43.85, the Youden index was at its maximum (YI = 0.297), showing that 43.85 was the best critical value for PNI (area under the curve (AUC) of the ROC was 0.644 with a sensitivity of 60.9% and a specificity of 65.6%). Hence, based on the best critical value of PNI, patients were divided the low-PNI group (<43.85) and the high-PNI group (≥ 43.85).

### Relationship Between PNI and Clinicopathological Characteristics

The relationship between PNI and clinicopathological characteristics of 165 patients enrolled in this study is summarized in Table 1. Among 165 patients, 34 (20.6%) were women and 131 (79.40%) were men. The mean age was 62.67 ± 7.95 years, with the age ranging from 44 to 85 years. The average PNI of 165 patients with ESCC was 46.68 ± 8.66. ROC curve showed that the best cutoff value was 43.85. All patients were divided into two groups: 72 patients (43.6%) were in the low PNI group (<43.85), while 93 patients (56.4%) were in the high PNI group (≥ 43.85). Our study showed that PNI value was associated with tumor size (*p* = 0.042), T classification (*p* = 0.002) and pathological stage (*p* = 0.014).

**Table 1:** Relationships between prognostic nutritional index (PNI) and clinicopathological characteristics in 165 patients with esophageal squamous cell carcinoma (ESCC).

**Characteristic**	**Total patients**	**PNI**		
		**PNI <43.5**	**PNI ≥43.5**	***P*-value**
		**(*n* = 72)**	**(*n* = 93)**	
Male	131	61	70	0.137
Female	34	11	23	
Age (years)		62.97 ± 8.00	62.44 ± 7.96	0.809
Tumor diameter (cm)		4.66 ± 1.87	3.87 ± 1.34	0.042
Tumor location				0.175
Upper	23	13	10	
Middle	93	35	58	
Lower	49	24	25	
Differentiation				0.668
Well	10	3	7	
Moderate	126	56	70	
Poor	29	13	16	
T classification				0.002
T1	35	8	27	
T2	26	7	19	
T3	93	50	43	
T4	11	7	4	
Lymph node metastasis				0.051
N0	80	30	50	
N1	43	16	27	
N2	25	16	9	
N3	17	10	7	
Pathological stage				0.014
I	30	7	23	
II	61	25	36	
III	74	40	34	

### PNI and Overall Survival

**Figure 2:**
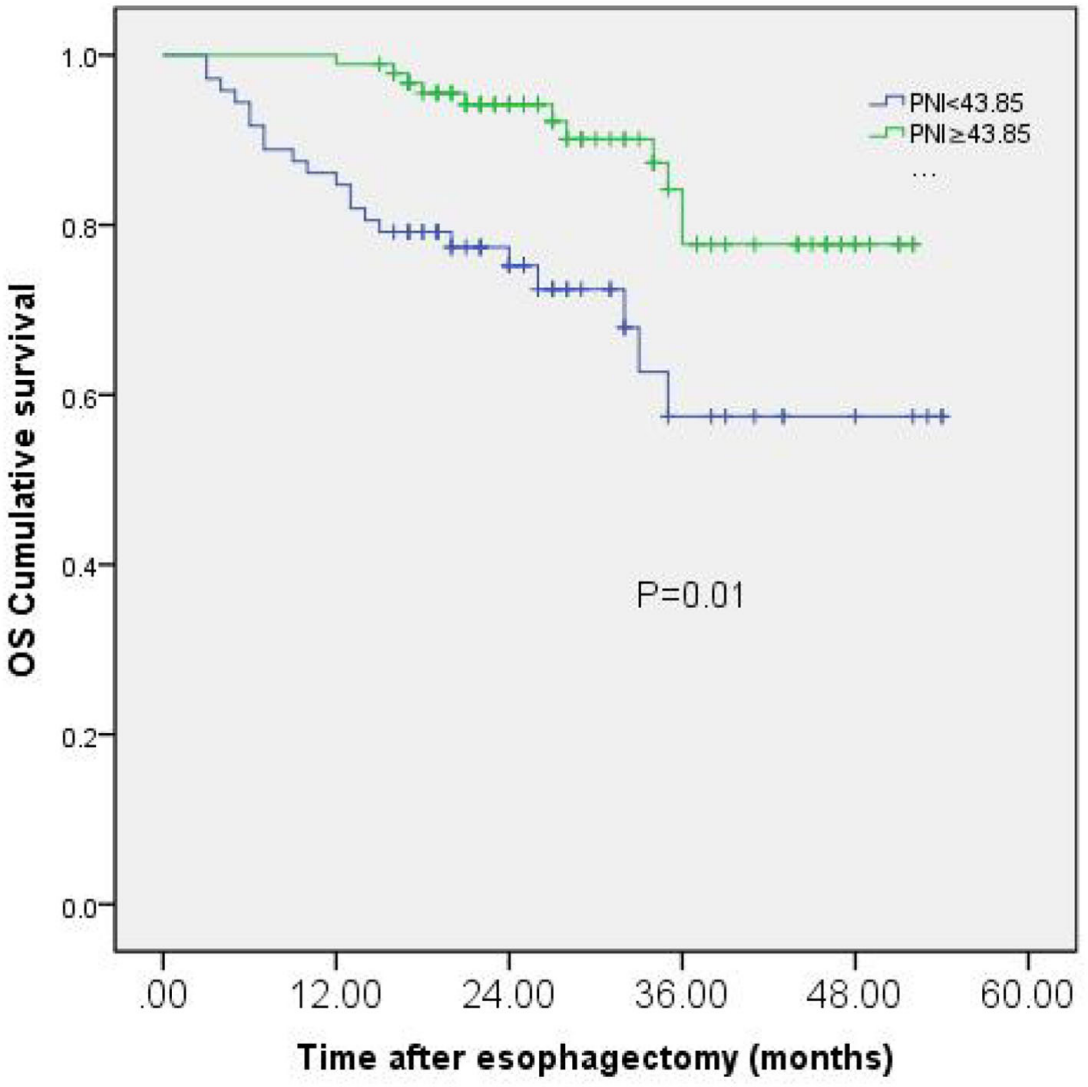
Kaplan-Meier curves of OS based on PNI group in 165 patients with esophageal squamous cell carcinoma (ESCC).

Finally, 165 patients were followed up and analyzed in our study. During the last follow-up date of September 2021, 133 patients (80.6%) were alive, while cancer-related death occurred in 32 patients. The Kaplan-Meier analysis and the log-rank test showed that patients with low PNI had a significantly worse prognosis in OS than those with high PNI (*p* = 0.01). To all patients, three-year OS rates were 57.5% in the low PNI group, while 77.7% in the high PNI group (Figure 2).

### Multivariate Analyses of Independent Prognostic Factors

Among 165 patients, univariate analysis showed that advanced pathological stage (*p* = 0.045), large tumor length (*p* < 0.001), and low PNI (*p* = 0.003) were significant risk factors for shorter OS. Multivariate analysis showed that tumor length (*P* = 0.008) and PNI (*P* = 0.017) were independent prognostic factors in patients with resectable ESCC (Table 2).

**Table 2:** Prognostic factors for overall survival (OS) in patients with ESCC.

**Variables**	**Patients (*n* = 165)**	**Category**	**Univariate**			**Multivariate**		
			**HR**	**95% CI**	***p* value**	**HR**	**95% CI**	***p* value**
Gender	131/34	Female/male	0.388	0.118–1.273	0.118			
Age	98/67	<65/≥65	1.015	0.973–1.060	0.486			
Pathological stage	91/74	I, II/ III	1.698	1.103–2.848	0.045			
Tumor length	59/106	<3 cm/≥3 cm	1.416	1.171–1.712	<0.001	1.313	1.072–1.608	0.008
PNI	72/93	<43.5/≥43.5	0.934	0.893–0.976	0.003	0.948	0.907–0.991	0.017

*HR, hazard ratio; CI, confidence interval*.

## Discussion

Until now, ESCC treatment strategies included surgery, chemotherapy, and radiation (11). Despite advances in the treatment of ESCC, radical surgical operation remains the modality choice of treatment, but the rate of postoperative recurrence rate is still high (12). Although tumor, nodes, and metastases (TNM) stage, tumor diameter, and lymph node metastasis can evaluate the prognosis of esophageal cancer, the predictive value is still limited for a long time. Therefore, it will become more and more important to find a simple, reliable, and repeatable factor that can accurately predict a patient's prognosis for ESCC. Accumulating studies (13–15) have demonstrated that the nutritional status and immune function are related to the occurrence and development of malignant cancers, while serum protein and lymphocyte count can reflect the nutritional status of the body. PNI index is a nutritional evaluation index that was put forward by Japanese scholar Onodera (16), which has been widely applied to evaluate the prognosis of cancer (17–19). Until now, there are few studies focused on the relationship between PNI and OS in resectable ESCC. Therefore, this study conducted a retrospective analysis to evaluate the prognostic value of PNI in resectable ESCC.

Prognostic nutritional index involves the values of serum albumin and peripheral blood lymphocyte count two parameters, which are routinely measured in clinical practice, particularly before surgical operation. Serum albumin is produced by hepatocytes, which is an important component of the plasma, and its level can reflect the body's nutritional status (20). Recently, several studies have shown that low serum albumin is a risk factor for malignant tumor prognosis (21–23), although it alone is not sufficient and accurate to predict the final outcome in cancer patients. Another calculated element of PNI is the blood lymphocyte count. Lymphocytes are one of the fundamental components of cell-mediated immunity with inhibitory effects on the proliferation and invasion of tumor cells *via* cytokine-mediated cytotoxicity (24, 25). Lymphocyte is an important part of the body's immunity, in which low lymphocyte indicates that the body's immunity is not good or there is a disorder, thereby making the prognosis of patients worse (26). Therefore, the decrease of PNI reflects the decreased inhibition of inflammatory response and tumor cell invasion, thus affecting the prognosis of tumor patients. However, the mechanism of PNI influencing the prognosis of tumor patients is not clear.

In our present study, we found that PNI, tumor length, and T-stage and pathological stage were related to the prognosis of patients with resectable ESCC. Multivariate analysis showed that tumor length and PNI were independent prognostic factors in patients with resectable ESCC. Patients with low PNI have significantly decreased OS in comparison to those with high PNI. These results suggest that PNI is a simple and useful predictive marker for the overall survival time in patients with radical esophagectomy.

The best critical cutoff value of PNI in predicting OS in patients with malignant tumors is still controversial, and previous studies showed various cutoff values for PNI (27–29). Therefore, another aim of our study was to propose and validate an optimal cutoff value which can predict OS with better accuracy in ESCC. The best cut-off value of PNI in our study was selected by drawing an ROC curve, which used OS time as the endpoint, and then calculating the maximum Youden index, which represented the best sensitivity and accuracy and has a good clinical practicability. However, the cutoff value for PNI in our study seemed to be lower than those reported in lung cancer and gliomas (30, 31). The possible reason may be that due to the location of esophageal cancer, most patients have some degree of swallowing difficulty, which results in poor nutritional status.

Finally, several limitations were considered in our study. First, this was a retrospective study that included a limited number of patients, which may lead to a selection bias. Second, due to the follow-up time, we lack the 5-year survival rate, and only had 3-year survival rate. Therefore, our conclusions may be strengthened by further exploration. On the other way, the long data collection time in this retrospective analysis and advances in surgical technology during this period may influence the clinical outcome.

## Conclusion

The present study demonstrated that the PNI is a simple and useful predictive marker of the OS time in patients with radical esophagectomy. PNI can be routinely calculated in patients with ESCC before surgery to help clinicians develop effective measures for early intervention.

## Data Availability Statement

The original contributions presented in the study are included in the article/Supplementary Material, further inquiries can be directed to the corresponding authors.

## Ethics Statement

The studies involving human participants were reviewed and approved by Cancer Hospital of Shantou University Medical College. Written informed consent for participation was not required for this study in accordance with the national legislation and the institutional requirements.

## Author Contributions

ZZ and HZ designed the study. ZZ was the main author of the manuscript and they performed data extraction and writing. HC supervised the project, assisted with the statistical analysis, and interpretation of the results. The paper was written by ZZ. All authors contributed to the article and approved the submitted version.

## Funding

This work was supported by the Science and Technology Special Fund of Guangdong Province of China (190829105556145) and Fujian Provincial Department of Science and Technology of China (grant no. 2020Y9070).

## Conflict of Interest

The authors declare that the research was conducted in the absence of any commercial or financial relationships that could be construed as a potential conflict of interest.

## Publisher's Note

All claims expressed in this article are solely those of the authors and do not necessarily represent those of their affiliated organizations, or those of the publisher, the editors and the reviewers. Any product that may be evaluated in this article, or claim that may be made by its manufacturer, is not guaranteed or endorsed by the publisher.
